# Exploring sex-specific hematological changes and their impact on quality of life in patients with prolactinoma

**DOI:** 10.1007/s11102-024-01493-x

**Published:** 2025-02-03

**Authors:** Mario Detomas, Timo Deutschbein, Pasquale Dolce, Yvonne Möhres, Martin Fassnacht, Barbara Altieri

**Affiliations:** 1 Department of Internal Medicine I, Division of Endocrinology and Diabetes, University Hospital, University of Würzburg, Würzburg, Germany; 2 Department of Endocrinology, William Harvey Research Institute, Barts and the London School of Medicine, Queen Mary University of London, London, UK; 3 Medicover Oldenburg MVZ, Oldenburg, Germany; 4 Department of Translational Medical Science, University of Naples Federico II, Naples, Italy; 5 Central Laboratory, University Hospital Würzburg, Würzburg, Germany

**Keywords:** Prolactin, Hemoglobin, Hypogonadism, Men, Women, Anemia

## Abstract

**Context:**

Despite prolactin´s (PRL) role in stimulating hematopoiesis, anemia is commonly observed in men with macroprolactinomas. However, hematological changes in men with microprolactinomas and women with prolactinomas remain unexplored, and the impact of erythropoietic alterations on quality of life (QoL) is still unclear.

**Objective:**

To explore sex-related changes in red blood cell (RBC) parameters and their potential impact on QoL at initial diagnosis of prolactinoma and after normalization of PRL under dopamine agonists.

**Design:**

Retrospective, monocentric study involving 205 patients with prolactinoma (127 women, 62%). The SF-36 QoL questionnaire was administered to 57 women and 34 men.

**Results:**

In women, no significant changes in RBC parameters were observed at diagnosis or after PRL normalization, regardless the adenoma size. Conversely, men with microprolactinoma showed a significant increase in hematocrit (HCT) and hemoglobin (Hb) levels after PRL normalization (median HCT 42.3 vs.44.0%; Hb 14.5 vs. 15.1 g/dL; both *p* < 0.005). Men with macroprolactinoma exhibited similar improvements (HCT 40.2 vs. 43.9%; Hb 14.0 vs. 15.1 g/dL; both *p* < 0.0001). In men, hypogonadism was observed in 73% of patients at baseline, and in 11% after PRL normalization. In male patients where SF-36 was administered at diagnosis and after PRL normalization, energy improvement was observed (median 50 vs. 60, *p* < 0.05). While changes in Hb and HCT were not significantly impacting the QoL of women and men, persistence of hypogonadism after PRL normalization, negatively impacted all the QoL scores of men.

**Conclusion:**

Patients with prolactinoma show sex-dependent changes in RBC parameters. Unlike women, men exhibit decreased HCT and Hb levels irrespective of adenoma size. Of note, the failure to recover from hypogonadism significantly affected the QoL of men.

**Supplementary Information:**

The online version contains supplementary material available at 10.1007/s11102-024-01493-x.

## Introduction

Prolactinomas are prolactin (PRL)-secreting pituitary adenomas derived from lactotroph cells and constitute the majority of hormone-secreting intra-sellar tumors in both women and men [1]. Like other pituitary adenomas, prolactinomas are categorized by size: microprolactinomas (less than 10 mm), macroprolactinomas (10 mm or larger). While microprolactinomas are more frequently observed in women, macroprolactinomas are more prevalent in men [1].

Regardless of size, prolactinomas are usually associated with secondary hypogonadism. In women, a hyperprolactinemia-induced estrogen deficiency results in menstrual irregularities, while in men, low testosterone levels can lead to impaired sexual function [1]. Additionally, prolactinomas may cause not only mastodynia and galactorrhea but also impaired mental health, thereby adversely affecting quality of life (QoL) in affected patients [2, 3].

It has been demonstrated that PRL can stimulate human hematopoietic CD34 + progenitor cells [4] and that treatment with drug inducing PRL release (e.g. metoclopramide) improved the hematocrit in subgroups of patients with anemia [5]. However, although the positive effect of PRL on erythropoiesis was also reported in other studies [6, 7], the exact mechanism behind this mechanism remains unclear. Considering that patients with prolactinoma often present secondary hypogonadism, and that estrogen [8, 9] and testosterone [10, 11] are well known to have a stimulating effect on erythropoiesis, hematological variations are expected in patients with prolactinoma. Indeed, anemia has been described in men with macroprolactinoma [12–14]. To date, however, it remains unclear whether similar erythropoietic alterations occur also in men with microprolactinoma or women with prolactinoma. Moreover, a possible correlation between variations in red blood cell (RBC) parameters and diminished QoL in patients with prolactinoma has to be assumed but has not been investigated in detail so far.

This study assessed potential sex-dependent differences in RBC parameters, their impact on QoL, and their potential reversibility during remission under medical treatment with dopamine agonists in a large cohort of patients with prolactinoma. Moreover, the impact on RBC parameters of hypogonadism as well as the deficit of the other pituitary hormones before and after dopamine agonist treatment was also considered.

## Subjects and methods

### Study design and population

Retrospective single-center analysis of patients with prolactinoma treated at the Division of Endocrinology and Diabetes of the University Hospital Würzburg between January 2007 and June 2023. Diagnosis of prolactinoma was performed in the case of hyperprolactinemia, presence of a pituitary adenoma and presence of symptoms related to hyperprolactinemia (e.g. mastodynia, galactorrhea and hypogonadism).

All patients with RBC parameters collected at the time of the initial diagnosis of the prolactinoma were considered eligible. Pathological results were identified according to age- and sex-specific range levels for RBC parameters [15, 16].

Patients were excluded if one of the following conditions was present during an interval of 4 weeks before blood sampling: (1) supplementation with iron, vitamin B12, and/or folate, (2) piles or gastric ulcera, (3) hematological disases, (4) malignant disease, (5) renal disease (GFR < 60 ml/min/1.73 m^2^) [17], and/or (6) administration of chemotherapy or other potentially hematotoxic drugs.

RBC parameters were analyzed both before and after normalization of PRL levels during treatment with dopamine agonists, after a therapy of at least 3 months. To assess the impact of hematological changes on patients’ QoL, RBC parameters set in context to results from Short Form Health Survey (SF-36) questionnaires which were completed by a subset of patients.

Patients provided written informed consent to participate in the clinical registry Network of Excellence for Neuroendocrine Tumors (NeoExNET), which was approved by the local Ethics Committee of the University Hospital Würzburg (reference number 85/12).

### Hormonal analysis

The analysis of PRL and other hormones was performed with commercially available analytical procedures (until July 2022, the Immulite system (Siemens) was applied; afterwards the analyses were conducted with the Cobas e 402 apparatus (Roche)).

ACTH deficiency was diagnosed when morning serum cortisol levels (between 08:00–09:00 h) were low (< 5 µg/dL) and/or cortisol peaked below 18 µg/L after stimulation with synthetic ACTH (250 µg iv) [18]. Central hypothyroidism was diagnosed when a subnormal serum free T4 (FT4) level (< 10 pmol/L) was associated with a low or low-normal TSH level (local reference range 0.3–5 mU/L) [19].

Adequacy of thyroid hormone replacement in primary hypothyroidism was assessed if TSH was in normal range. In secondary hypothyroidism, adequacy was determined if free T4 was in the mid-to-upper reference range. In the case of insufficient thyroid hormone replacement or diagnosis of hypothyroidism, were reported in the text as overt hypothyroidism. In postmenopausal women, overt hypogonadism was identified when serum LH and/or FSH levels were inappropriately low for their age. In premenopausal women, overt hypogonadism was diagnosed in the presence of amenorrhea or oligomenorrhea, when baseline gonadotropin levels were low or at the lower end of the normal range (normal LH: 2–15 IU/L; normal FSH: 2–10 IU/L), along with persistently low estradiol levels (< 30 pg/mL) [19]. In men, overt hypogonadism was diagnosed when both testosterone and gonadotropin levels were below the normal age-specific reference ranges [20]. Normalization of hypogonadism was defined as gonadal and gonadotropin hormone levels falling within age- and sex-appropriate reference ranges, accompanied by the resolution of previous clinical symptoms, such as menstrual irregularities or erectile dysfunction.

Growth hormone (GH) deficiency was confirmed with a GHRH-arginine test. In detail, the test was considered pathological if GH was below 4 µg/l after stimulation. Results were adjusted according to the body mass index (as reported in the guidelines) [21].

### Red blood cells analysis

Analysis of the RBC parameters, including hematocrit (HCT), RBC count, mean corpuscular volume (MCV), hemoglobin (Hb), mean corpuscular Hb (MCH), and mean corpuscular Hb concentration (MCHC), was performed with GenS Beckman (until 2009), Sysmex XE-2100 (from 2009 until 2017), and Sysmex XN-9000 (from 2017 onwards). According to local comparisons and published data [22, 23].

### Analysis of the quality of life

Quality of life (QoL) was evaluated using the widely-used, self-administered generic health questionnaires SF-36 in its German version [24]. The SF-36 questionnaire includes statements designed to assess eight specific health domains: physical functioning, physical pain, role limitations due to physical health problems, role limitations due to personal or emotional problems, emotional well-being, social functioning, energy/fatigue, and general health perceptions. Each statement is answered on a five-point Likert scale, with higher scores indicating better health. Scores for each domain are calculated by averaging the responses, with overall scores ranging from 0 to 100.

### Statistical analysis

Categorical variables were expressed as frequencies and percentage, and comparison between groups were performed using the Chi-square (χ^2^) test. Quantitative variables were tested for Gaussian distribution with the Shapiro-Wilk test. Normally distributed variables were presented as mean ± standard deviation (SD), while not-normally distributed variables were shown as median and interquartile range (IQR). Comparison between groups were performed using Student´s T-tests or ANOVA followed by Tukey *post-hoc* test for normally distributed quantitative variables and using Mann-Whitney U test or Kruskal–Wallis test followed by Dunn’s *post-hoc* test for not-normally distributed quantitative variables. For the matched paired analysis, paired t-test or Wilcoxon matched-pairs signed rank test were performed.

To evaluate the relation between various variables and red blood cell parameters as well as QoL scores, simple linear regression analyses were initially performed. Subsequently, variables with a p-value < 0.10 were included in multiple linear regression models to identify independent predictors. Of note, for the variable “difference in PRL from baseline” (calculated as the difference between prolactin levels under dopamine agonists and at diagnosis), the coefficient is expressed per 100 µg/L change in prolactin.

Statistical significance for all tests was set to α = 0.05. Statistical analysis was performed with SPSS version 29 (IBM Corporation, Armonk, NY, USA) and GraphPad Prism version 9 (GraphPad Software, San Diego, CA, USA).

## Results

### Hematological changes in the female population

A total of 127 women with prolactinoma (102 with micro- and 25 with macroprolactinoma) were identified. Data on the female study population at the time of diagnosis of prolactinoma is reported in Table 1. At initial diagnosis, no significant differences in RBC parameters were identified between women with microprolactinoma and macroprolactinoma. Moreover, considering the entire female population, no significant correlation between prolactin levels and any RBC parameters was observed.

**Table 1:** Hormonal and hematological data of the study population at the time of diagnosis of micro- and macroprolactinoma

	Women (*n* = 127)		*p*-value	Men (*n* = 78)		*p*-value	*p*-value (all groups)
	Micro- (*n* = 102)	Macro- (*n* = 25)		Micro- (*n* = 23)	Macro- (*n* = 55)		
Age, years (IQR)	33 (19)	34 (33)	> 0.999	42 (18)	48 (23)	> 0.999	0.0002
Smoking, n (%)	8 (8)	2 (8)	> 0.999	4 (17)	8 (15)	0.741	0.107
**Hormonal data**							
Prolactin levels, µg/l (IQR)	71 (60)	321 (547)	**< 0.0001**	72 (87)	1037 (2440)	< 0.0001	**< 0.0001**
Hypogonadism, n (%)	61 (60)	19 (76)	0.168	14 (61)	43 (78)	0.161	0.0774
ACTH deficiency, n (%)	0 (0)	5 (20)	**0.0002**	0 (0)	18 (33)	**0.0009**	**< 0.0001**
TSH deficiency, n (%)	0 (0)	7 (28)	**0.0012**	0 (0)	20 (36)	**0.0028**	**< 0.0001**
GH deficiency, n (%) *	0 (0)	3 (12)	**0.0069**	0 (0)	6 (11)	0.171	**0.0007**
**Red blood cell parameters**							
Red blood cell count, n*10^6^/µl (IQR)	4.6 (0.5)	4.5 (0.5)	0.518	4.9 (0.4)	4.7 (0.6)	0.125	**< 0.0001**
Hematocrit, % (IQR)	40.1 (3.3)	38.3 (2.4)	0.296	42.8 (2.7)	40.2 (4.0)	**0.0122**	**< 0.0001**
Hemoglobin, g/dl (IQR)	13.6 (1.9)	13.1 (1.0)	0.736	14.7 (1.2)	14.0 (2.0)	**0.0337**	**< 0.0001**
Number of patients with anemia, n (%)	6 (6)	1 (4)	> 0.999	5 (22)	30 (55)	**0.0119**	**< 0.0001**
Mean corpuscular volume, fl. (IQR)	87.1 (5.2)	86.3 (4.6)	> 0.999	87.3 (5.5)	86.7 (3.1)	> 0.999	0.720
MCH, pg (IQR)	29.6 (1.9)	29.7 (1.4)	> 0.999	30.1 (2.0)	29.7 (1.8)	> 0.999	0.665
MCHC, g/dl (IQR)	33.9 (1.1)	34.0 (1.1)	> 0.999	34.3 (0.7)	34.2 (1.4)	> 0.999	0.115

*tested in 12 (6%) of the patients

Hypogonadism was reported in 61 (60%) patients with microprolactinoma and 19 (76%) patients with macroprolactinoma, whereas deficiency of the other pituitary hormones was found only in a subset of patients with macroprolactinoma (Table 1). Serum iron, ferritin, transferrin, folate and vitamin B12 levels were available in 9% of the patients and are reported in Supplementary Table 1. The number of patients under hormone replacement therapy at the time of diagnosis is reported in Supplementary Table 2.

Data on 65 (51%) patients (48 micro- and 17 macroprolactinomas at initial diagnosis) with prolactin normalization under dopamine agonists (cabergoline, *n* = 57; bromocriptine, *n* = 6; quinagolide, *n* = 2) were available. Median time from diagnosis of prolactinoma to RBC parameter analysis after PRL normalization was 24 months (IQR 24). No difference in any RBC parameters was observed in patients with micro- and macroprolactinoma between the time of diagnosis and the time of prolactin normalization (Table 2). In line of normalization of PRL levels, hypogonadism significantly improved in 23 out 28 patients with microprolactinoma and in 8 out 11 patients with macroprolactinoma (Supplementary Table 3). In patients with macroprolactinoma, ACTH and TSH deficiency improved in 2 out 3 and 2 out 5 cases, respectively, at time of PRL normalization, whereas GH was still present in 2 patients (Supplementary Table 3).

**Table 2:** Red blood cell parameters in women at the time of diagnosis of prolactinoma and after prolactin normalization

	Microprolactinoma (*n* = 48)			Macroprolactinoma (*n* = 17)		
	At diagnosis	After PRL normalization	*p*-value	At diagnosis	After PRL normalization	*p*-value
Red blood cell count, n*10^6^/µl (IQR)	4.7 (0.4)	4.6 (0.4)	0.714	4.4 (0.3)	4.5 (0.4)	0.219
Hematocrit, % (IQR)	40.3 (2.7)	39.9 (2.5)	0.963	38.0 (3.6)	39.1 (3.5)	0.423
Hemoglobin, g/dl (IQR)	13.7 (1.3)	13.5 (1.5)	0.888	13.0 (0.9)	13.1 (1.1)	0.311
Number of patients with anemia, n (%)	5 (10)	4 (8)	> 0.999	0 (0)	2 (12)	0.484
Mean corpuscular volume, fl. (IQR)	86.7 (3.8)	87.6 (5.3)	0.692	86.9 (4.7)	85.9 (4.5)	0.426
MCH, pg (IQR)	29.5 (1.9)	29.6 (2.0)	0.538	29.7 (0.9)	29.5 (0.7)	0.141
MCHC, g/dl (IQR)	33.9 (1.5)	34.0 (1.3)	0.946	34.1 (1.2)	34.1 (1.0)	0.463

To assess the impact of overt hypogonadism, overt hypothyroidism (primary or secondary), prolactin change from diagnosis, age, smoking, and hormone replacement therapies (glucocorticoids, thyroid hormones, estrogen) on RBC, HCT and Hb at PRL normalization, a multivariable regression was performed. Under dopamine agonists, untreated hypogonadism was observed in 5 (8%) women, and untreated/inadequately treated hypothyroidism in 6 patients (10%). The mean prolactin change from baseline was − 3.23 ± 8.28 µg/L, and the mean age was 40 ± 17 years. Smoking was reported by 5 (8%) women. One patient was on glucocorticoid replacement, 12 on thyroid hormone, and 3 on estrogen; none were on GH replacement. As shown in Table 3, age minimally but significantly positively influenced RBC and Hb increase.

**Table 3:** Logistic regression analysis of hormonal and clinical predictors for hematological changes in women following prolactin normalization

	Univariate analysis		Multivariable analysis	
	b	*p*-value	b	*p*-value
**Changes of RBC count from baseline**				
Overt hypogonadism	0.011 (-0.239; 0.260)	0.932	-	-
Overt hypothyroidism	-0.251 (-0.514; 0.012)	0.061	-0.171 (-0.438; 0.096)	0.206
Difference of PRL from baseline (µg/l)	-0.006 (-0.14; 0.003)	0.198	-	-
Age ( years)	0.007 (0.001–0.010)	0.012	0.005 (0.001; 0.100)	**0.035**
Smoking	-0.115 (-0.471; 0.241)	0.520	-	-
Glucocorticoid replacement	0.532 (-0.047; 1.110)	0.171	-	-
Thyroid hormone replacement	0.062 (-0.120; 0.243)	0.497	-	-
Estrogen replacement	-0.083 (-0.359; 0.193)	0.548	-	-
**Changes of HCT count from baseline**				
Overt hypogonadism	0.007 (-2.223; 2.237)	0.995	-	-
Overt hypothyroidism	-2.662 (-4.989; -0.334)	0.007	-2.030 (-4.406; 0.346)	0.093
Difference of PRL from baseline (µg/l)	-0.038 (-0.117; 0.041)	0.240	-	-
Age ( years)	0.049 (0.009; 0.089)	0.018	0.039 (-0.002; 0.080)	0.062
Smoking	-0.240 (-3.416; 2.937)	0.880	-	-
Glucocorticoid replacement	3.933 (-1.359; 9.224)	0.142		
Thyroid hormone replacement	0.540 (-1.104; 2.185)	0.659		
Estrogen replacement	-1.139 (-3.627; 1.349)	0.363		
**Changes of Hb from baseline**				
Overt hypogonadism	0.138 (-0.572; 0.845)	0.702	-	-
Overt hypothyroidism	-0.830 (-1.571; -0.89)	0.029	-0.596 (-1.345; 0.153)	0.117
Difference of PRL from baseline (µg/l)	-0.012 (-0.037; 0.013)	0.353	-	-
Age ( years)	0.017 (0.005; 0.030)	0.008	0.14 (0.001; 0.027)	**0.029**
Smoking	-0.013 (-0.993; 0.967)	0.979	-	-
Glucocorticoid replacement	1.275 (-0.388; 2.937)	0.130		
Thyroid hormone replacement	0.107 (-0.412; 0.626)	0.680		
Estrogen replacement	-0.536 (-1.311; 0.239)	0.171		

### Hematological changes in the male population

The male population comprised 78 patients (23 with micro- and 55 with macroprolactinoma). Hormonal and hematological data of these patients at baseline is reported in Table 1.

Serum iron, ferritin, transferrin, folate and vitamin B12 levels were available in 13% of the patients and are reported in Supplementary Table 1. At initial diagnosis, patients with microprolactinoma showed higher HCT (median 42.8 [2.7] vs. 40.2 [4.0] %, *p* = 0.0122) and Hb (14.7 [1.2] g/dl vs. 14.0 [2.0], *p* = 0.0337) than those with macroprolactinoma (Table 1). Patients with microprolactinoma had higher testosterone levels (median 1.7 [1.3] vs. 1.2 [0.8] µg/l, *p* = 0.006) than patients with macroprolactinoma. Of note, hypogonadism was observed in 61% of patients with microprolactinoma and 78% with macroprolactinoma (*p* = 0.161). In patients with macroprolactinoma, ACTH and TSH deficiency was found in approximately one third of cases, while GH deficiency was confirmed in 11% of the affected patients (Table 1). The number of patients under hormone replacement therapy at the time of diagnosis is reported in Supplementary Table 2.

Data on 54 patients (69%) with normalized prolactin levels under dopamine agonists (cabergoline, *n* = 47; bromocriptine, *n* = 6; quinagolide, *n* = 1) were available, including 13 micro- and 41 macroprolactinomas. Median time from diagnosis of prolactinoma to RBC parameter analysis after PRL normalization was 12 (IQR 24) months. After PRL normalization, HCT and Hb levels significantly improved in men with microprolactinoma (median HCT 42.3 [1.8] vs. 44.0 [1.6] % and median Hb 14.5 [0.6] vs. 15.1 [0.7] g/dl, both *p* < 0.05) as well as in those with macroprolactinoma (median HCT 40.2 [3.7] vs. 43.9 [2.2] % and median Hb 13.9 [2.0] vs. 15.1 [1.5] g/dl, both *p* < 0.001) (Table 4). Furthermore, in men with macroprolactinoma a higher RBC count (4.7 [0.4] vs. 5.0 [0.6] n*10^6^/µl, *p* < 0.0001) was identified after PRL normalization than at the time of diagnosis. Of note, 16 out 24 patients with macroprolactinoma recovered from anemia under dopamine agonists (Table 4). Hypogonadism improved after PRL normalization in 5 out 6 patients with microprolactinoma and 28 out 33 patients with macroprolactinoma (Supplementary Table 4). Furthermore, in patients with macroprolactinoma, TSH deficiency improved in 9 out 15 cases (*p* = 0.0228) and ACTH deficiency improved

in 7 out 16 cases after PRL normalization. No improvement in terms of GH deficiency was reported under dopamine agonists in patients with available data (Supplementary Table 4).

**Table 4:** Red blood cell parameters in men at the time of diagnosis of prolactinoma and after prolactin normalization

	Microprolactinoma (*n* = 13)			Macroprolactinoma (*n* = 41)		
	At diagnosis	After PRL normalization	*p*-value	At diagnosis	After PRL normalization	*p*-value
Red blood cell count, n*10^6^/µl (IQR)	4.8 (0.3)	4.9 (0.5)	0.184	4.7 (0.4)	5.0 (0.6)	**< 0.0001**
Hematocrit, % (IQR)	42.3 (1.8)	44.0 (1.6)	**0.0327**	40.2 (3.7)	43.9 (2.2)	**< 0.0001**
Hemoglobin, g/dl (IQR)	14.5 (0.6)	15.1 (0.7)	**0.0244**	13.9 (2.0)	15.1 (1.5)	**< 0.0001**
Number of patients with anemia, n (%)	3 (23)	0 (0)	0.065	24 (59)	8 (20)	**0.0003**
Mean corpuscular volume, fl. (IQR)	87.3 (3.2)	88.2 (7.6)	0.384	86.2 (2.8)	85.8 (4.5)	0.782
MCH, pg (IQR)	30.2 (2.0)	30.3 (1.9)	0.186	29.4 (1.7)	29.6 (1.4)	0.378
MCHC, g/dl (IQR)	34.2 (1.2)	34.5 (1.7)	0.906	34.2 (1.2)	34.6 (1.8)	0.297

Overt hypogonadism and hypothyroidism (primary or secondary) were observed in 4 (7%) male patients after PRL normalization. The mean change in PRL levels from baseline was − 16.44 ± 26.29 µg/L. The mean age of the patients was 48 ± 15 years. Smoking was reported by 7 (13%) men. 9 patients were on glucocorticoid replacement, 3 on adequate thyroid hormone replacement and 2 on testosterone replacement therapy; none were on GH replacement. As shown in Table 5, presence of hypogonadism significantly negatively impacted RBC count, HCT and Hb increase from levels at diagnosis.

**Table 5:** Logistic regression analysis of hormonal and clinical predictors for hematological changes in men following prolactin normalization

	Univariate analysis		Multivariable analysis	
	b	*p*-value	b	*p*-value
**Changes of RBC count from baseline**				
Overt hypogonadism	-0.471 (-0.718; -0.225)	**< 0.0001**	-	-
Overt hypothyroidism	0.091 (-0.233; 0.416)	0.574	-	-
Difference of PRL from baseline (µg/l)	-0.002 (-0.005; 0.002)	0.422	-	-
Age (years)	-0.001 (-0.008; 0.006)	0.706	-	-
Smoking	0.272 (-0.082; 0.626)	0.127	-	-
Glucocorticoid replacement	0.177 (-0.181; 0.536)	0.324	-	-
Thyroid hormone replacement	-0.038 (-0.255; 0.178)	0.722	-	-
Testosterone replacement	0.103 (-0.309; 0.515)	0.617	-	-
**Changes of HCT from baseline**				
Overt hypogonadism	-4.117 (-5.915; 2.319)	**< 0.0001**	-	-
Overt hypothyroidism	1.050 (-1.424; 3.524)	0.398	-	-
Difference of PRL from baseline (µg/l)	-0.015 (-0.44; 0.015)	0.325	-	-
Age (years)	0.007 (-0.047-0.062)	0.788	-	-
Smoking	1.651 (-1.201; 4.504)	0.248	-	-
Glucocorticoid replacement	2.333 (-0.525; 5.192)	0.107	-	-
Thyroid hormone replacement	0.264 (-1.496; 2.024)	0.764	-	-
Testosterone replacement	1.488 (-1.842; 4.818)	0.373	-	-
**Changes of Hb from baseline**				
Overt hypogonadism	-1.682 (-2.307; -1.057)	**< 0.0001**	-	-
Overt hypothyroidism	0.404 (-0.500; 1.308)	0.374	-	-
Difference of PRL from baseline (µg/l)	-0.007 (-0.018; 0.003)	0.177	-	-
Age (years)	0.004 (-0.015; 0.024)	0.660	-	-
Smoking	0.503 (-0.580; 1.586)	0.353	-	-
Glucocorticoid replacement	0.403 (-0.736; 0.1543)	0.479	-	-
Thyroid hormone replacement	0.106 (-0.579; 0.792)	0.756	-	-
Testosterone replacement	0.056 (-1.252; 1.356)	0.932	-	-

### Sex-specific quality of life and impact of hematological changes

In total, 101 SF-36 QoL questionnaires were completed by 51 women and 34 men.

**Fig. 1:**
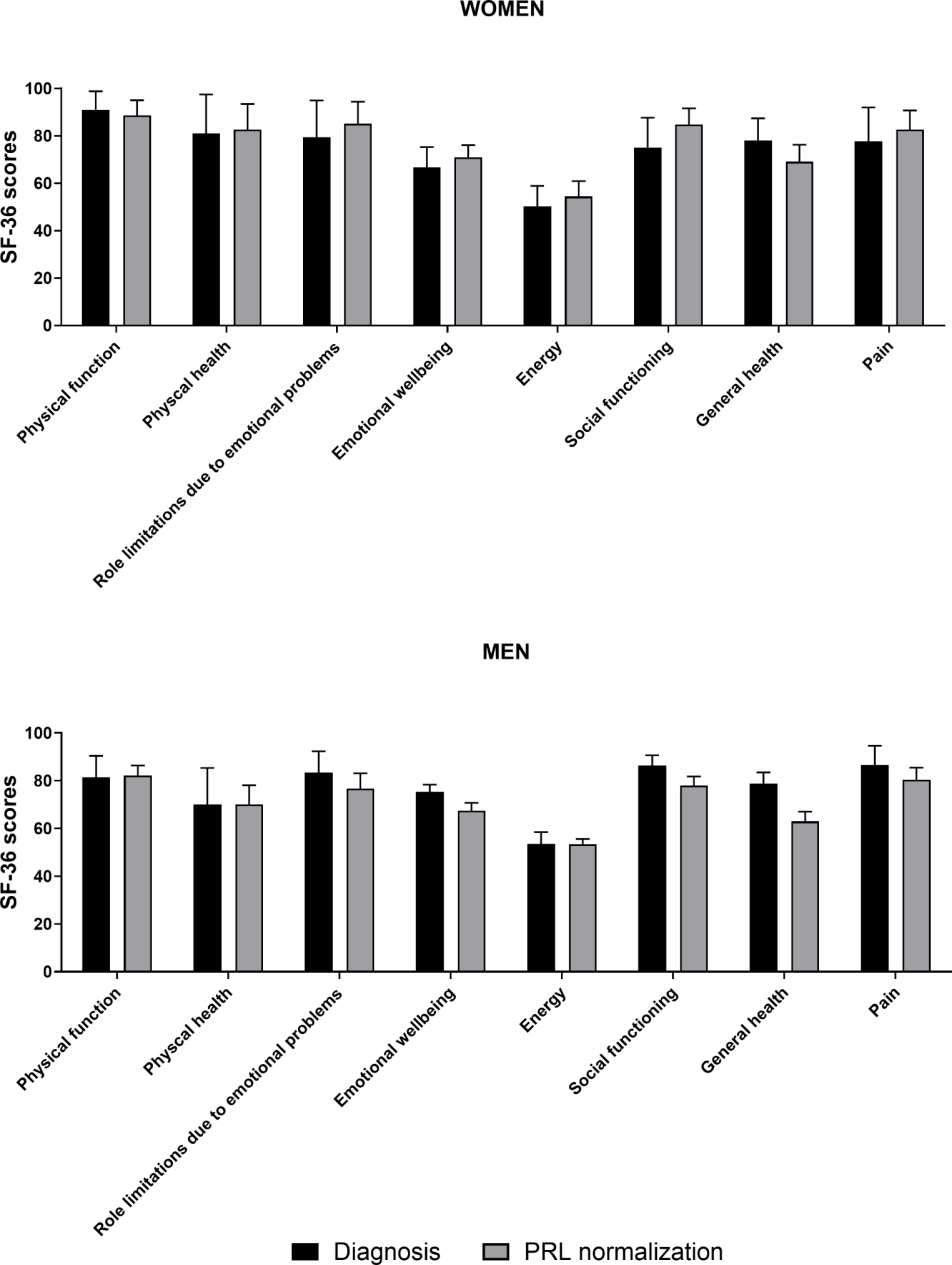
SF-36 scores at initial diagnosis and after prolactin normalization under dopamine agonists. Questionnaires were completed either at the time of the initial diagnosis (women, *n* = 21; man, *n* = 10) or after prolactin normalization (women, *n* = 40; man, *n* = 30). Results are reported as mean and 95%-confidence interval. No significant difference in any of the scores was found Abbreviation: PRL, prolactin

**Fig. 2:**
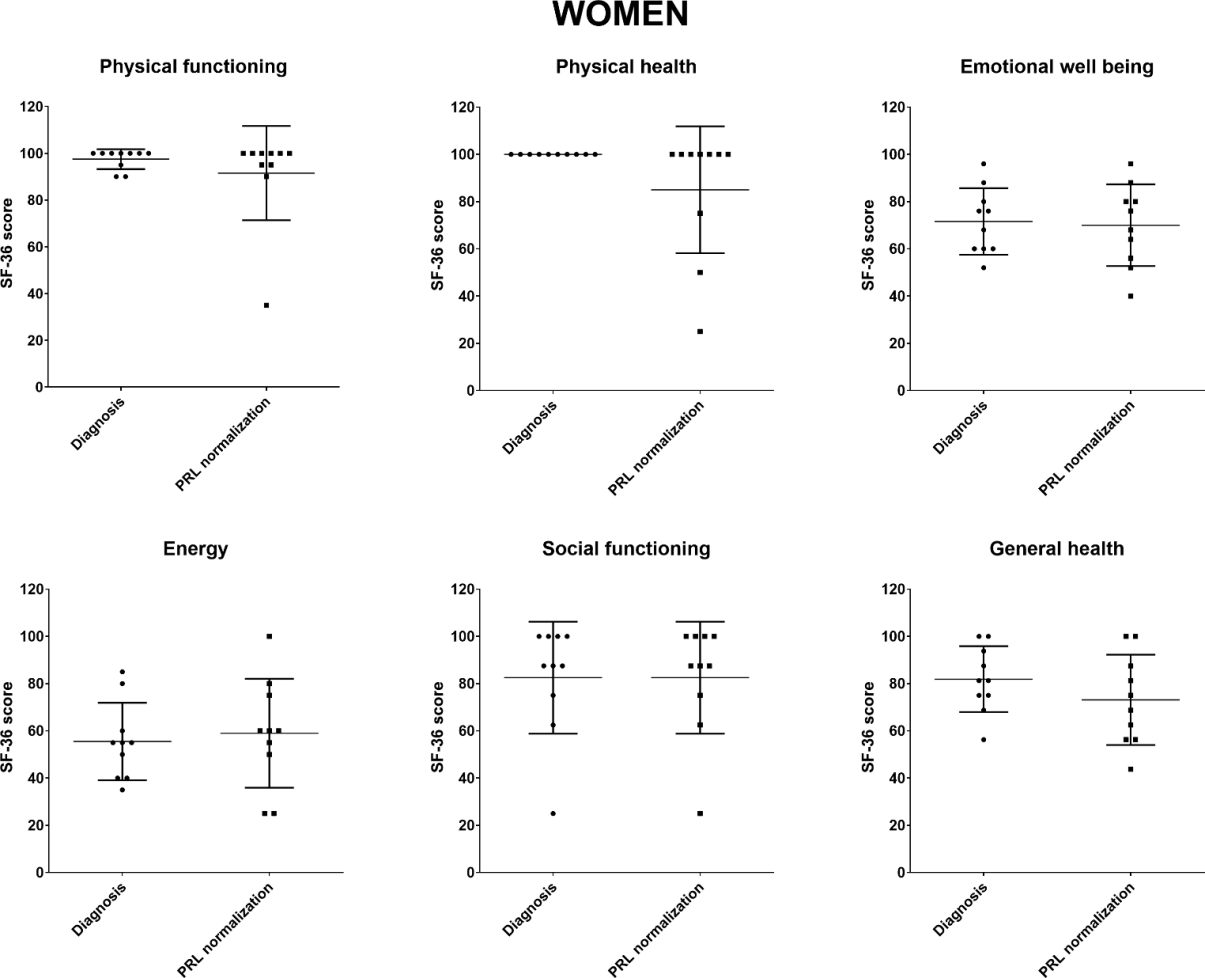
SF-36 quality of life scores of 10 women who completed the questionnaire both at initial diagnosis and after prolactin normalization under dopamine agonists. No significant difference in any of the scores was observed Abbreviation: PRL, prolactin

Among women, the SF-36 questionnaire was completed at the time of the initial diagnosis and/or after PRL normalization during medical therapy by 21 and 40 patients, respectively. As shown in Fig. 1, similar SF-36 scores were observed at initial diagnosis and after remission. Of note, 10 women completed the SF-36 questionnaire both at initial diagnosis and after dopamine-induced PRL normalization, with results indicating no significant differences in QoL scores between the two time points (Fig. 2). In these 10 patients, there were also no significant differences in terms of HCT and Hb at initial diagnosis and after PRL normalization (median HCT 39.6 [2.8] vs. 38.5 [2.9] %, *p* = 0.92; median Hb 13.6 [1.2] vs. 13.3 [0.8] g/dl, *p* = 0.57).

To assess the impact of overt hypogonadism and hypothyroidism (primary or secondary), PRL change from diagnosis, age, HCT and Hb changes from diagnosis on QoL scores after PRL normalization, a multivariable regression analysis was performed. Age was negatively impacting physical function (b= -0.493 [-0.907; -0.078], *p* = 0.021), emotional wellbeing (b= -0.389, [-0.737; -0.041], *p* = 0.029), (energy b= -0.420 [-0.833; -0.007] *p* = 0.047), general health (b= -0.767 [-1.197; -0.338], *p* < 0.0001). Of note, both HCT and Hb changes did not impact any QoL scores (Supplementary Table 5).

**Fig. 3:**
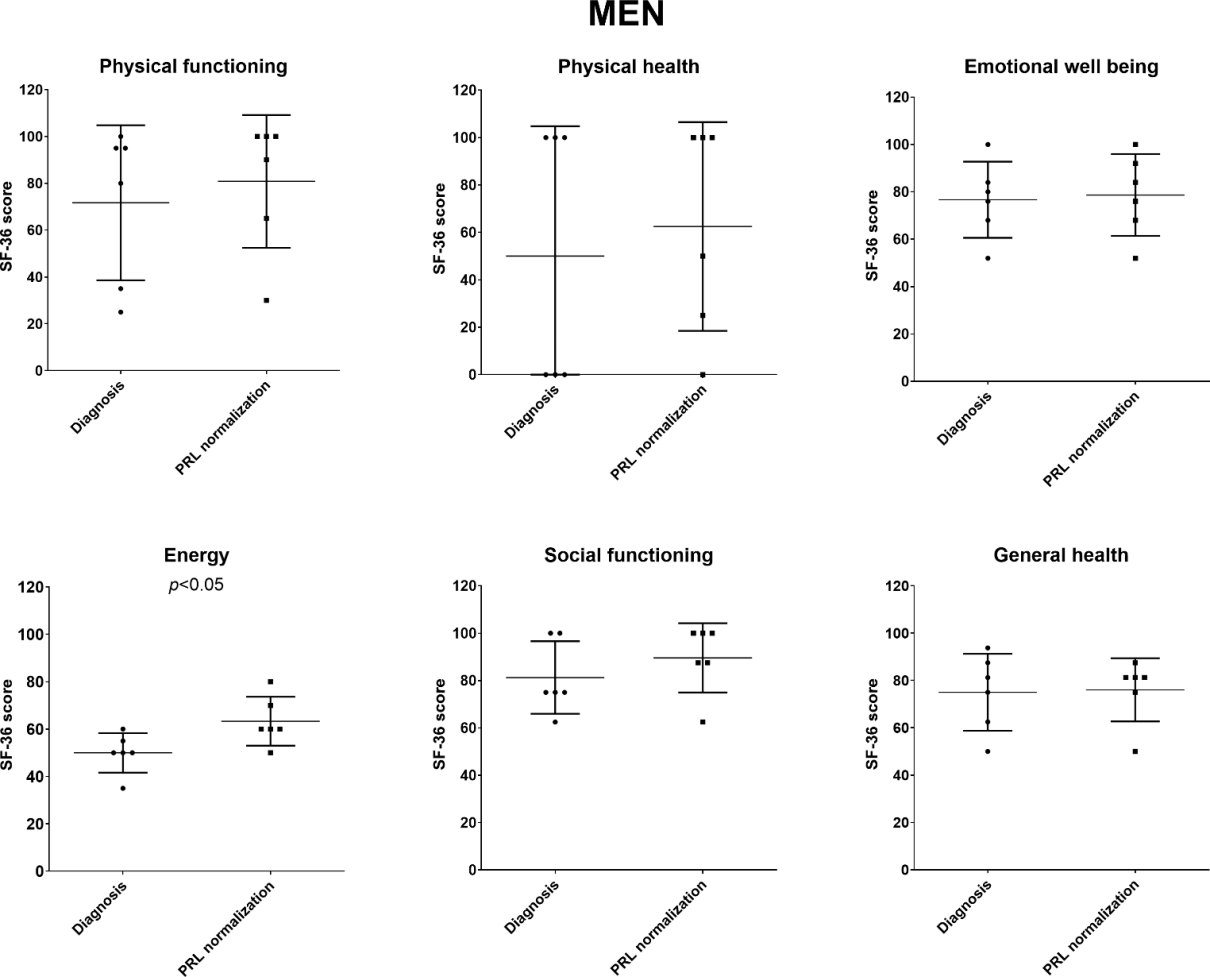
SF-36 quality of life scores of 6 men who completed the questionnaire both at initial diagnosis and after prolactin normalization under dopamine agonists. Over the time of treatment, significant differences in the energy score was observed Abbreviation: PRL, prolactin

Regarding the male study population, each one SF-36 questionnaire was completed either at initial diagnosis and/or after prolactin normalization under dopamine agonists by 10 and 30 patients, respectively. Mirroring the findings in women, there were no significant differences in the SF-36 scores between men at initial diagnosis and after PRL normalization (Fig. 1). Six men completed the questionnaire both at initial diagnosis and after PRL normalization during medical therapy. In these 6 patients, a significant improvement in energy scores between diagnosis and remission was observed (50 vs. 60, *p* = 0.022) (Fig. 3). Importantly, in these subgroup of patients, an increase of median testosterone levels from 1.1 (0.6) µg/l at the time of diagnosis to 2.8 (3.3) µg/l after PRL normalization (*p* = 0.031), as well as an increase in HCT (from 40.9 [3.3] to 44.0 [2.0] %) and Hb (from 14.1 [1.4] to 15.2 [0.9] g/dl) was detected (both *p* < 0.05).

Persistency of hypogonadism was significantly impairing all the QoL scores, while difference in PRL from diagnosis (b = 0.508 [0.159; 0.858], *p* = 0.007) and age (b= -1.166, [-2.238; -0.94], *p* = 0.035) were impacting respectively physical function and physical health (Supplementary Table 6).

## Discussion

Our large monocentric study is the first to analyze sex-specific differences in RBC parameters in patients with prolactinoma and to explore the potential impact of these hematological changes as well as of PRL and sex hormones changes on QoL. While women did not show significant changes in RBC parameters, men exhibited lower levels of HCT and Hb at initial diagnosis of prolactinoma, independently from the size of the adenoma. Notably, normalization of PRL and testosterone levels in men after treatment with dopamine agonists resulted in increased HCT and Hb. However, our data suggest how the changes in HCT and Hb did not affect the QoL of both women and men, while persistency of hypogonadism significantly impair the QoL of men after PRL normalization.

Considering that anemia has been reported in men with macroprolactinoma [12, 14], one of the main aims of this study was to determine whether similar alterations could be observed in women with prolactinoma. Despite hyperprolactinemia is frequently associated with hypogonadism, women with prolactinoma did not show any abnormalities in RBC parameters. This was a surprising result considering that estrogen typically plays an important role in iron homeostasis, with elevated estrogen levels being associated with increased systemic iron availability [9]. Additionally, estrogen has a positive effect on erythropoiesis and proliferation of hematopoietic stem cells in several physiological conditions [8, 25]. Although 63% of the women in our study suffered from secondary hypogonadism, anemia was observed in only 5% of the patients. A possible explanation for this is the compensatory effect of dehydroepiandrosterone (DHEAS), which in patients with prolactinoma is higher in women than in men [26]. In fact, it has been demonstrated that DHEAS could stimulate the erythropoiesis [27]. However, this remains speculative and requires confirmation through future studies, since the retrospective nature of the current study unfortunately does not allow us to perform this analysis.

Anemia has been previously described as a common feature in men with macroprolactinoma [13, 28] However, data on RBC changes in men with microprolactinoma were missing before our study. Similarly to what has previously been reported [13, 28], we identified at baseline anemia in 55% of men with macroprolactinoma but also in 22% of those with microprolactinoma. Moreover, different RBC parameters, including HCT and Hb significantly improved after PRL normalization with dopamine agonists treatment in both men with micro- and macroprolactinoma. Also, an increase of RBC count was observed in men with macroprolactinoma after PRL normalization. Elevated prolactin levels inhibit the activity of hypothalamic kisspeptin-producing neurons causing a decrease in gonadotropin-releasing hormone release and, consequently, a decrease in testosterone levels in men [29]. Therefore, the improvement in RBC parameters could be most likely attributed to the normalization of hypogonadism and the concurrent increase in testosterone levels, due to the normalization of PRL. Indeed, testosterone exerts a stimulatory effect on erythropoietin production and hematopoietic stem cells [11]. In addition to that, macroprolactinomas might affect erythropoiesis also by impairing normal pituitary gland function e.g. by their size [12]. For instance, GH, thyroid hormones and cortisol exert a stimulatory role on erythropoiesis through different mechanisms [23, 30, 31], and therefore patients with the respective hormonal deficiencies often present with anemia. Of note, persistent hypogonadism after PRL normalization showed a negative correlation with RBC, HCT, and Hb levels, whereas hypothyroidism and any hormonal replacement therapy had no significant impact on hematological parameters. Although these results were observed in a small cohort, they suggest how hypogonadism might affect the erythropoiesis more than other pituitary hormone deficiencies in men with prolactinoma, while it has not a significant impact on hematological parameters in women.

To identify the clinical relevance of these hematological changes and pituitary hormones following high PRL levels, we analyzed the QoL through the administration of the SF-36 questionnaires at time of the diagnosis of prolactinoma and after PRL normalization. In women, no significant changes in RBC parameters or QoL scores were observed at initial diagnosis or after PRL normalization. This suggests that the improvement in RBC after medical treatment with dopamine agonists might have no impact on the QoL in women with prolactinoma. Our results are in line with previous studies that underline a potential negative impact of dopamine agonists on QoL due to psychiatric treatment-related adverse events, including increased anxiety and depression [3, 32]. Of note, we identified in age the only variable significantly affecting QoL of women under dopamine agonists.

In contrast, a subgroup of men with prolactinomas who completed the questionnaire both at diagnosis and during dopamine agonist therapy showed improved energy scores following PRL normalization and resolution of hypogonadism. Considering that anemia might negatively affect the energy of individuals and that both HCT and Hb increased in these patients at follow-up, we would have expected that the increase in HCT and Hb was significantly correlating with the QoL of the patients with normalized PRL. However, the only variables that were affecting the QoL of men were hypogonadism, change in PRL levels from diagnosis and age. Interestingly, persistence of hypogonadism was negatively affecting all the QoL of men with normalized PRL, confirming the importance of testosterone in the QoL of men [33].

To our knowledge, we report for the first time a sexual dimorphism in the improvement of QoL in patients with prolactinoma treated with dopamine agonists. Furthermore, we investigated factors influencing the QoL of women and men with normalized PRL under dopamine agonists. However, the small number of patients completing the QoL questionnaire at both time of initial diagnosis and PRL normalization, limits the generalizability of these findings and future prospective studies with larger cohorts of men affected by prolactinoma are needed to confirm our results. Nevertheless, this study suggests that normalizing hypogonadism is important for improving the QoL and especially the energy of the affected patients.

Several limitations of this study must be acknowledged, including its retrospective nature, the small number of patients providing QoL questionnaire both at initial diagnosis and during treatment, and the limited number of patients tested for GH deficiency and with complete data on iron status, vitamin B12, and folate. Furthermore, the exact time of the menstrual cycle, that could have affected the hematological levels in women, was not available. Despite these limitations, our single-center study includes a large cohort of prolactinoma patients, with the male cohort being one of the largest reported in the literature. The homogenous diagnostic, therapeutic, and scientific workup of our patients certainly increases the robustness and reliability of our findings.

In conclusion, prolactinomas might be associated with lower levels of Hb and HCT in affected men, regardless of their adenoma size. The changes in RBC parameters are more pronounced in patients with macroadenomas and may be due not only to lower testosterone levels but also to concomitant hypopituitarism. Although this analysis was performed in a very small group, our results suggests that normalization of prolactin and testosterone in men, with consequent increase in Hb and HCT levels, is associated to an increase in energy and QoL. Of note, after PRL normalization, persistency of hypogonadism was negatively impacting the QoL of the patients. In women, hematological changes are less significant and seem not to impact QoL as much as in men.

## Electronic supplementary material

Below is the link to the electronic supplementary material.

Supplementary Material 1

Supplementary Material 2

Supplementary Material 3

Supplementary Material 4

Supplementary Material 5

Supplementary Material 6

Supplementary Material 7

## Data Availability

No datasets were generated or analysed during the current study.
