# Supplementary material for: Exploring sex-specific hematological changes and their impact on quality of life in patients with prolactinoma
Source: Pituitary. 2025 Feb 3;28(1):24. doi: 10.1007/s11102-024-01493-x (PMC11790753; doi:10.1007/s11102-024-01493-x)
Supplement: Supplementary file 2 — Supplementary Material 2 [file 11102_2024_1493_MOESM2_ESM.docx]

**Supplementary Table 1**. Available levels of iron, ferritin, transferrin, vitamin B12 and folic acid in the study population at the time of prolactinoma diagnosis.

|  | **Women** | | **Men** | | **p-value** |
| --- | --- | --- | --- | --- | --- |
|  | Microprolactinoma  (n=9) | Macroprolactinoma  (n=2) | Microprolactinoma  (n=3) | Macroprolactinoma  (n=7) |  |
| Iron, µg/dl (IQR) | 244 (43) | 281 (92) | 211 (4) | 264 (36) | 0.259 |
| Ferritin, µg/l (IQR) | 67 (57) | 57 (27) | 83 (203) | 122 (123) | 0.167 |
| Transferrin, mg/dl (IQR) | 244 (48) | 281 (194) | 211 (4) | 263 (36) | 0.165 |
| Vitamin B12 pg/ml (IQR) | 299 (530) | 318 (56) | 371 (155) | 334 (310) | 0.573 |
| Folic acid, ng/ml (IQR) | 9.0 (6.8) | 6.3 (3.4) | 4.0 (4.0) | 4.8 (23) | 0.068 |

Considering that the range values are the same in men and women, we performed a ANOVA analysis, considering the 4 groups.
