# Supplementary material for: Exploring sex-specific hematological changes and their impact on quality of life in patients with prolactinoma
Source: Pituitary. 2025 Feb 3;28(1):24. doi: 10.1007/s11102-024-01493-x (PMC11790753; doi:10.1007/s11102-024-01493-x)
Supplement: Supplementary file 3 — Supplementary Material 3 [file 11102_2024_1493_MOESM3_ESM.docx]

**Supplementary Table 2.** Number of patients under hormone replacement therapy at the time of diagnosis and at the time of PRL normalization.

| **Women** | | | | | | | | |
| --- | --- | --- | --- | --- | --- | --- | --- | --- |
|  | | Diagnosis of prolactinoma  (n=127) | | | | After prolactin normalization  (n=65) | | |
|  | | Microprolactinoma | | Macroprolactinoma | | Microprolactinoma | | Macroprolactinoma |
| Thyroid hormones substitution, n (%) | | 10 (8%) | | 1 (1%) | | 8 (12%) | | 4 (6%) |
| Glucocorticoids substitution , n (%) | | 0 | | 0 | | 0 | | 1 (2%) |
| Estrogens substitution , n (%) | | 10 (8%) | | 4 (3%) | | 1 (2%) | | 2 (3%) |
| Growth-hormone substitution, n (%) | | 0 | | 0 | | 0 | | 0 |
| **Men** | | | | | | | | |
|  | Diagnosis of prolactinoma  (n=78) | | | | After prolactin normalization  (n=54) | | | |
|  | Microprolactinoma | | Macroprolactinoma | | Microprolactinoma | | Macroprolactinoma | |
| Thyroid hormones substitution, n (%) | 1 (1%) | | 0 | | 1 (2%) | | 2 (4%) | |
| Glucocorticoids substitution, n (%) | 0 | | 0 | | 0 | | 9 (17%) | |
| Testosterone substitution, n (%) | 0 | | 2 (3%) | | 1 (2%) | | 1 (2%) | |
| Growth-hormone substitution, n (%) | 0 | | 0 | | 0 | | 0 | |
