# Supplementary material for: Exploring sex-specific hematological changes and their impact on quality of life in patients with prolactinoma
Source: Pituitary. 2025 Feb 3;28(1):24. doi: 10.1007/s11102-024-01493-x (PMC11790753; doi:10.1007/s11102-024-01493-x)
Supplement: Supplementary file 4 — Supplementary Material 4 [file 11102_2024_1493_MOESM4_ESM.docx]

**Supplementary Table 3.** Hormonal data of the female population before and after prolactin normalization

|  | **Microprolactinoma**  **(n=48)** | | | **Macroprolactinoma**  **(n=17)** | | |
| --- | --- | --- | --- | --- | --- | --- |
|  | **At diagnosis** | **After PRL normalization** | **p-value** | **At diagnosis** | **After PRL normalization** | **p-value** |
| Prolactin levels, µg/l (IQR) | 81 (85) | 14 (12) | **<0.0001** | 416 (804) | 8 (16) | **<0.0001** |
| Hypogonadism, n (%) | 28 (57) | 5 (10) | **<0.0001** | 11 (65) | 3 (18) | **0.0134** |
| ACTH deficiency, n (%) | 0 (0) | 0 (0) | - | 3(18) | 1 (6) | 0.337 |
| TSH deficiency, n (%) | 0 (0) | 0 (0) | - | 5 (29) | 3 (18) | 0.688 |
| GH deficiency, n (%) * | 0 (0) | 0 (0) | - | 2 (12) | 2 (12) | >0.999 |

*tested in 3 (5%) of the patients
