# Supplementary material for: Exploring sex-specific hematological changes and their impact on quality of life in patients with prolactinoma
Source: Pituitary. 2025 Feb 3;28(1):24. doi: 10.1007/s11102-024-01493-x (PMC11790753; doi:10.1007/s11102-024-01493-x)
Supplement: Supplementary file 5 — Supplementary Material 5 [file 11102_2024_1493_MOESM5_ESM.docx]

**Supplementary Table 4.** Hormonal data of the male population before and after prolactin normalization

|  | **Microprolactinoma**  **(n=13)** | | | **Macroprolactinoma**  **(n= 41)** | | |
| --- | --- | --- | --- | --- | --- | --- |
|  | **At diagnosis** | **After PRL normalization** | **p-value** | **At diagnosis** | **After PRL normalization** | **p-value** |
| Prolactin levels, µg/l (IQR) | 45 (82) | 7 (12) | **0.0002** | 1043 (2534) | 9 (17) | **<0.0001** |
| Hypogonadism, n (%) | 6 (46) | 1 (8) | **0.0271** | 33 (80) | 5 (12) | **<0.0001** |
| ACTH deficiency, n (%) | 0 (0) | 0 (0) | - | 16 (39) | 9 (22) | 0.093 |
| TSH deficiency, n (%) | 0 (0) | 0 (0) | - | 15 (37) | 6 (15) | **0.0228** |
| GH deficiency, n (%) | 0 (0) | 0 (0) | - | 4 (10) | 4 (10) | >0.999 |

*tested in 4 (10%) of the patients
