# Supplementary material for: Exploring sex-specific hematological changes and their impact on quality of life in patients with prolactinoma
Source: Pituitary. 2025 Feb 3;28(1):24. doi: 10.1007/s11102-024-01493-x (PMC11790753; doi:10.1007/s11102-024-01493-x)
Supplement: Supplementary file 6 — Supplementary Material 6 [file 11102_2024_1493_MOESM6_ESM.docx]

**Supplementary Table 5.** Regression analysis of quality of life scores in relation to hematological, hormonal, and clinical parameters in women following prolactin normalization.

| ***WOMEN*** | | | | |
| --- | --- | --- | --- | --- |
|  | ***Coefficient univariate analysis*** | ***Univariate analysis***  ***p-value*** | ***Coefficient multivariate analysis*** | ***Multivariate analysis***  ***p-value*** |
| ***Physical function*** | | | | |
| *Overt hypogonadism* | *-5.305 (-27.903; 17.293)* | *0.638* | *-* | ***-*** |
| *Overt hypothyroidism* | *6.726*  *(-15.629; 29.081)* | *0.547* | *-* | *-* |
| *Difference of PRL from diagnosis (µg/l)* | *0.039*  *(-1.428; 1.507)* | *0.957* | *-* | ***-*** |
| *Age (years)* | *-0.697*  *(-1.067; -0.326)* | *<0.0001* | *-0.493*  *(-0.907; -0.078)* | ***0.021*** |
| *Difference in Hb from diagnosis (g/dl)* | *-3.262*  *(-10.540; 1.984)* | *0.097* | *-9.985*  *(-26.266; 6.705)* | *0.121* |
| *Difference in HCT from diagnosis (%)* | *-2.264*  *(-4.754; 0.231)* | *0.074* | *3.646*  *(-1.420; 8.712)* | *0.152* |
| ***Physical health*** | | | | |
| *Overt hypogonadism* | *-31.707*  *(-69.925; 6.511)* | *0.102* | *-* | ***-*** |
| *Overt hypothyroidism* | *22.619*  *(-15.838; 61.076)* | *0.242* | *-* | *-* |
| *Difference of PRL from diagnosis (µg/l)* | *-0.373*  *(-3.299; 2.556)* | *0.797* | *-* | ***-*** |
| *Age (years)* | *-0.939*  *(-1.624; -0.253)* | *0.008* | *-1.166*  *(-1.615; 0.154)* | *0.097* |
| *Difference in Hb from diagnosis (g/dl)* | *-19.300*  *(-34.049; -4.550)* | *0.081* | *-33.620*  *(-68.961; 1.142)* | *0.075* |
| *Difference in HCT from diagnosis (%)* | *-4.269*  *(-9.272; 0.737)* | *0.092* | *6.944*  *(-3.873; 17.761)* | *0.200* |
| ***Role limitations due to emotional problems*** | | | | |
| *Overt hypogonadism* | *19.512*  *(-14.429; 53.545)* | *0.253* | *-* | ***-*** |
| *Overt hypothyroidism* | *19.841*  *(-13.725; 53.408)* | *0.240* | *-* | *-* |
| *Difference of PRL from diagnosis (µg/l)* | *-0.809*  *(-3.360; 1.742)* | *0.522* | *-* | *-* |
| *Age (years)* | *-0.543*  *(-1.170; 0.083)* | *0.088* | *-* | *-* |
| *Difference in Hb from diagnosis (g/dl)* | *1.480*  *(-12.841; 15.801)* | *0.834* | *-* | *-* |
| *Difference in HCT from diagnosis (%)* | *1.918*  *(-2.623; 6.460)* | *0.396* | *-* | *-* |
| ***Emotional wellbeing*** | | | | |
| *Overt hypogonadism* | *-4.756*  *(-24.247; 14.734)* | *0.625* | *-* | ***-*** |
| *Overt hypothyroidism* | *6.429*  *(-12.817; 25.674)* | *0.504* | *-* | *-* |
| *Difference of PRL from diagnosis (µg/l)* | *0.145*  *(-1.333; 1.622)* | *0.843* | *-* | *-* |
| *Age (years)* | *-0.389*  *(-0.737; -0.041)* | ***0.029*** | *-* | ***-*** |
| *Difference in Hb from diagnosis (g/dl)* | *-4.073*  *(-12.189; 4.043)* | *0.314* | *-* | *-* |
| *Difference in HCT from diagnosis (%)* | *-0.477*  *(-3.117; 2.168)* | *0.715* | *-* | *-* |
| ***Energy*** | | | | |
| *Overt hypogonadism* | *-16.768*  *(-39.150; 5.614)* | *0.138* | *-* | ***-*** |
| *Overt hypothyroidism* | *8.214*  *(-14.404; 30.833)* | *0.468* | *-* | *-* |
| *Difference of PRL from diagnosis (µg/l)* | *-1.365*  *(-3.066; 0.336)* | *0.112* | *-* | *-* |
| *Age (years)* | *-0.420*  *(-0.833; -0.007)* | ***0.047*** | *-* | ***-*** |
| *Difference in Hb from diagnosis (g/dl)* | *-3.208*  *(-13.033; 6.617)* | *0.510* | *-* | *-* |
| *Difference in HCT from diagnosis (%)* | *-0.484*  *(-3.652; 2.684)* | *0.758* | *-* | *-* |
| ***Social functioning*** | | | | |
| *Overt hypogonadism* | *-11.966*  *(-37.217; 13.285)* | *0.345* | *-* | ***-*** |
| *Overt hypothyroidism* | *15.030*  *(-9.883; 39.943)* | *0.231* | *-* | *-* |
| *Difference of PRL from diagnosis (µg/l)* | *-0.413*  *(-2.347; 1.521)* | *0.666* | *-* | *-* |
| *Age (years)* | *-0.427*  *(-0.891; 0.036)* | *0.070* | *-* | *-* |
| *Difference in Hb from diagnosis (g/dl)* | *-5.550*  *(-16.182; 5.082)* | *0.295* | *-* | ***-*** |
| *Difference in HCT from diagnosis (%)* | *-0.217*  *(-3.687; 3.253)* | *0.899* | *-* | *-* |
| ***General health*** | | | | |
| *Overt hypogonadism* | *-5.335*  *(-31.341; 20.671)* | *0.681* | *-* | *-* |
| *Overt hypothyroidism* | *-6.897*  *(-54.147; 40.345)* | *0.702* | *-* | *-* |
| *Difference of PRL from diagnosis (µg/l)* | *-0.202*  *(-2.105; 1.701)* | *0.830* | *-* | *-* |
| *Age (years)* | *-0.767*  *(-1.197; -0.338)* | ***<0.0001*** | *-* | ***-*** |
| *Difference in Hb from diagnosis (g/dl)* | *-6.839*  *(-17.167; 3.489)* | *0.187* | *-* | *-* |
| *Difference in HCT from diagnosis (%)* | *-1.313*  *(-4.686; 2.061)* | *0.434* | *-* | *-* |
| ***Pain*** | | | | |
| *Overt hypogonadism* | *-10.930*  *(-40.780; 18.920)* | *0.464* | *-* | ***-*** |
| *Overt hypothyroidism* | *4.583*  *(-25.076; 34.242)* | *0.757* | *-* | *-* |
| *Difference of PRL from diagnosis (µg/l)* | *-0.668*  *(-2.895; 1.558)* | *0.545* | *-* | *-* |
| *Age (years)* | *-0.395*  *(-0.946; 0.157)* | *0.156* | *-* | *-* |
| *Difference in Hb from diagnosis (g/dl)* | *-5.246*  *(-17.596; 7.107)* | *0.393* | *-* | *-* |
| *Difference in HCT from diagnosis (%)* | *-0.009*  *(-4.018; 4.000)* | *0.996* | *-* | *-* |

Overt hypogonadism was observed in 4 (9%) women, and overt hypothyroidism in 4 patients (9%).

The mean prolactin change from baseline was -2.30 ± 4.82 µg/L, the mean age was 39 ± 15 years.

Hb mean value was 0.23± 0.85 g/dl, mean HCT was 0.79 ± 2.65 %.
