# Supplementary material for: Exploring sex-specific hematological changes and their impact on quality of life in patients with prolactinoma
Source: Pituitary. 2025 Feb 3;28(1):24. doi: 10.1007/s11102-024-01493-x (PMC11790753; doi:10.1007/s11102-024-01493-x)
Supplement: Supplementary file 7 — Supplementary Material 7 [file 11102_2024_1493_MOESM7_ESM.docx]

**Supplementary Table 6.** Regression analysis of quality of life scores in relation to hematological, hormonal, and clinical parameters in men following prolactin normalization.

| ***MEN*** | | | | |
| --- | --- | --- | --- | --- |
|  | ***Coefficient univariate analysis*** | ***Univariate analysis***  ***p-value*** | ***Coefficient multivariate analysis*** | ***Multivariate analysis***  ***p-value*** |
| ***Physical function*** | | | | |
| *Overt hypogonadism* | *-19.687 (-39.900; 0.525)* | *0.056* | *-23.714*  *-44.391; -3.037)* | ***0.027*** |
| *Overt hypothyroidism* | *-22.931*  *(-70.466; 24.604)* | *0.332* | *-* | *-* |
| *Difference of PRL from diagnosis (µg/l)* | *0.411*  *(0.060; 0.761)* | *0.023* | *0.508*  *(0.159; 0.858)* | ***0.007*** |
| *Age (years)* | *-0.687*  *(-1.219; -0.156)* | *0.013* | *-0.512*  *(-1.055; 0.031)* | *0.063* |
| *Difference in Hb from diagnosis (g/dl)* | *5.700*  *(-0.119; 11.519)* | *0.055* | *2.677*  *(-8.809; 14.163)* | *0.630* |
| *Difference in HCT from diagnosis (%)* | *2.648*  *(0.014; 5.283)* | *0.049* | *-0.798*  *(-5.778; 4.183)* | *0.740* |
| ***Physical health*** | | | | |
| *Overt hypogonadism* | *-48.875*  *(-81.349; -10.376)* | *0.010* | *-43.245*  *(85.880; -0.611)* | ***0.047*** |
| *Overt hypothyroidism* | *-20.690*  *(-114.143; 72.763)* | *0.654* | *-* | *-* |
| *Difference of PRL from diagnosis (µg/l)* | *0.323*  *(-0.413; 1.059)* | *0.376* | *-* | ***-*** |
| *Age (years)* | *-1.194*  *(-2.250; -0.138)* | *0.028* | *-1.166*  *(-2.238; -0.94)* | ***0.035*** |
| *Difference in Hb from diagnosis (g/dl)* | *15.068*  *(4.500; 25.635)* | *0.007* | *7.381*  *(-14.961; 29.723)* | *0.498* |
| *Difference in HCT from diagnosis (%)* | *6.198*  *(1.292; 11.105)* | *0.015* | *-3.500*  *(-13.494; 6.495)* | *0.473* |
| ***Role limitations due to emotional problems*** | | | | |
| *Overt hypogonadism* | *-54.167*  *(-77.939; -30.395)* | *<0.001* | *-42.610*  *(-73.870; -11.351)* | ***0.010*** |
| *Overt hypothyroidism* | *-10.345*  *(-84.827; 64.137)* | *0.778* | *-* | *-* |
| *Difference of PRL from diagnosis (µg/l)* | *-0.305*  *(-0.887; 0.277)* | *0.292* | *-* | *-* |
| *Age (years)* | *-0.469*  *(-1.368; 0.429)* | *0.294* | *-* | *-* |
| *Difference in Hb from diagnosis (g/dl)* | *13.666*  *(5.656; 21.675)* | *0.002* | *8.486*  *(-7.865; 24.838)* | *0.292* |
| *Difference in HCT from diagnosis (%)* | *0.490*  *(1.014; 8.825)* | *0.015* | *1.203*  *(-8.564; 6.158)* | *0.737* |
| ***Emotional wellbeing*** | | | | |
| *Overt hypogonadism* | *-24.500*  *(-39.008; -9.992)* | *0.002* | *-26.567* | ***0.011*** |
| *Overt hypothyroidism* | *-29.138*  *(-64.362; 6.086)* | *0.101* | *-* | *-* |
| *Difference of PRL from diagnosis (µg/l)* | *-0.107*  *(-0.415; 0.200)* | *0.480* | *-* | *-* |
| *Age (years)* | *-0.156*  *(-0.632; 0.318)* | *0.503* | *-* | *-* |
| *Difference in Hb from diagnosis (g/dl)* | *4.405*  *(-0.310; 9.121)* | *0.066* | *-1.550*  *(-11.963; 8.863)* | *0.759* |
| *Difference in HCT from diagnosis (%)* | *1.967*  *(-0.174; 4.108)* | *0.070* | *0.230*  *(-4.457; 4.917)* | *0.919* |
| ***Energy*** | | | | |
| *Overt hypogonadism* | *-23.438*  *(-35.334; -11.541)* | *<0.0001* | *-22.448*  *(-38.776; -6.121)* | ***0.010*** |
| *Overt hypothyroidism* | *-29.138*  *(-64.362; 6.086)* | *0.110* | *-* | *-* |
| *Difference of PRL from diagnosis (µg/l)* | *-0.098*  *(-0.390; 0.194)* | *0.497* | *-* | *-* |
| *Age (years)* | *-0.103*  *(-0.556; 0.350)* | *0.644* | *-* | *-* |
| *Difference in Hb from diagnosis (g/dl)* | *6.079*  *(1.941; 10.216)* | *0.005* | *-1.526*  *(-7.015; 10.067)* | *0.713* |
| *Difference in HCT from diagnosis (%)* | *2.381*  *(0.563; 4.199)* | *0.012* | *-0.471*  *(-4.316; 3.374)* | *0.801* |
| ***Social functioning*** | | | | |
| *Overt hypogonadism* | *-27.344*  *(-42.934; -11.753)* | *0.001* | *-24.219*  *(-40.259; -8.178)* | ***0.005*** |
| *Overt hypothyroidism* | *-41.810*  *(-83.150; -0.471)* | *0.048* | *-25.000*  *(-62.841; 12.841)* | *0.184* |
| *Difference of PRL from diagnosis (µg/l)* | *-0.106*  *(-0.457; 0.245)* | *0.541* | *-* | *-* |
| *Age (years)* | *-0.333*  *(-0.863; 0.198)* | *0.209* | *-* | *-* |
| *Difference in Hb from diagnosis (g/dl)* | *4.245*  *(-1.226; 9.717)* | *0.123* | *-* | ***-*** |
| *Difference in HCT from diagnosis (%)* | *1.862*  *(-0.648; 4.373)* | *0.140* | *-* | *-* |
| ***General health*** | | | | |
| *Overt hypogonadism* | *-22.266*  *(-40.878; -3.656)* | *0.021* | *-21.547*  *(-47.087; 4.174)* | *0.096* |
| *Overt hypothyroidism* | *-6.897*  *(-54.147; 40.345)* | *0.767* | *-* | *-* |
| *Difference of PRL from diagnosis (µg/l)* | *-0.106*  *(-0.480; 0.269)* | *0.576* | *-* | *-* |
| *Age (years)* | *-0.410*  *(-0.970; 0.150)* | *0.147* | *-* | ***-*** |
| *Difference in Hb from diagnosis (g/dl)* | *5.387*  *(-0.336; 11.109)* | *0.064* | *0.177*  *(-13.220; 13.584)* | *0.978* |
| *Difference in HCT from diagnosis (%)* | *2.442*  *(-0.172; 5.056)* | *0.066* | *0.108*  *(-5.928; 6.143)* | *0.971* |
| ***Pain*** | | | | |
| *Overt hypogonadism* | *-22.969*  *(-43.346; -2.592)* | *0.029* | *-21.176*  *(-40.861; -1.491)* | ***0.048*** |
| *Overt hypothyroidism* | *7.328*  *(-50.588; 65.243)* | *0.797* | *-* | *-* |
| *Difference of PRL from diagnosis (µg/l)* | *-0.169*  *(-0.626; 0.287)* | *0.454* | *-* | *-* |
| *Age (years)* | *-0.664*  *(-1.329; 0.001)* | *0.050* | *-0.512*  *(-1.128; 0.104)* | *0.098* |
| *Difference in Hb from diagnosis (g/dl)* | *4.733*  *(-2.500; 11.967)* | *0.191* | *-* | *-* |
| *Difference in HCT from diagnosis (%)* | *2.661*  *(-0.546; 5.868)* | *0.103* | *-* | *-* |

Overt hypogonadism was observed in 4 (13%) male patients after PRL normalization, while overt hypothyroidism in 3 (10%). The mean prolactin change from baseline was -11.02 ± 22.9 µg/L, the mean age was 47 ± 15 years. Hb mean value was 0.60 ± 1.42 g/dl, mean HCT was 1.50 ± 3.2 %.
